# Solution landscape of reaction-diffusion systems reveals a nonlinear mechanism and spatial robustness of pattern formation

**DOI:** 10.1016/j.fmre.2025.10.006

**Published:** 2025-10-27

**Authors:** Shuonan Wu, Bing Yu, Yuhai Tu, Lei Zhang

**Affiliations:** a School of Mathematical Sciences, Peking University, Beijing 100871, China; b Center for Computational Biology & Center for Computational Neuroscience, Flatiron Institute, New York, NY 10010, USA; c Beijing International Center for Mathematical Research, Center for Quantitative Biology, Center for Machine Learning Research, Peking University, Beijing 100871, China

**Keywords:** Turing pattern, Pattern formation, Turing instability, Solution landscape, Saddle dynamics, Robust positioning

## Abstract

Spontaneous pattern formation in homogeneous systems is ubiquitous in nature. Although Turing demonstrated that spatial patterns can emerge in reaction-diffusion (RD) systems when the homogeneous state becomes linearly unstable, it remains unclear whether Turing mechanism is the only route for pattern formation. Here, we develop an efficient algorithm to systematically construct the solution landscape to find all steady-state solutions connected to a homogeneous state. By applying our method to generic RD models, we find that stable spatial patterns can emerge via saddle-node bifurcations before the onset of Turing instability, and reveal a general nonlinear mechanism that increases the parameter space over which pattern formation occurs in the RD systems. Furthermore, by using a generalized action in the functional space based on large deviation theory, our method is extended to evaluate stability of spatial patterns against noise. Applying this general approach in a three-species RD model, we show that though formation of Turing patterns only requires two chemical species, the third species is critical for stabilizing patterns against strong intrinsic noise in small biochemical systems.

## Highlights

• We propose a generic and efficient numerical algorithm to systematically construct the complete solution landscape of reaction-diffusion systems.
• We demonstrate that Turing instability is not the prerequisite to generate stable spatial patterns, uncovering a generic nonlinear mechanism that is beyond the original Turing mechanism.
• We elucidate the underlying mechanism for the robust positioning of spatial patterns in a reversible three-species RD system with intrinsic noise.

## Graphical abstract

**Figure:**
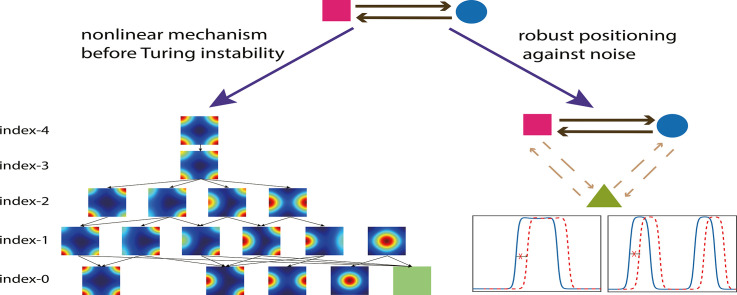

## 1 Introduction

**Fig. 1:**
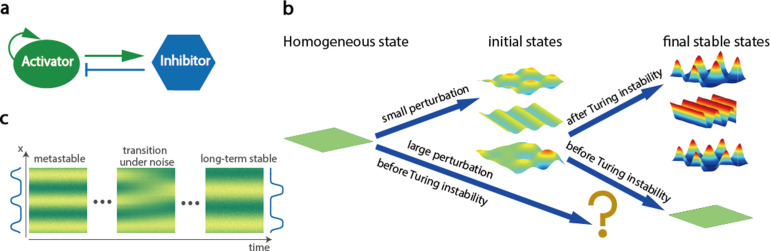
(a) Illustration of a two-species RD system for Turing pattern. (b) Turing instability indicates small perturbation of an unstable homogeneous state can generate stable inhomogeneous patterns. But, before Turing instability occurs, can large perturbation of a stable homogeneous state develop stable spatial patterns? (c) One-dimensional example of pattern transition under noise.

Alan Turing, in his seminal study on morphogenesis [1], suggested that biological pattern formation can be understood by reaction-diffusion (RD) dynamics [2], [3], [4]. In the simplest scenario, nonlinear chemical reactions between two diffusive chemical species, a short-range activator and a long-range inhibitor (as illustrated in Fig. 1a), can spontaneously generate spatial patterns, so-called Turing patterns [5], [6], [7], [8]. As shown in Fig. 1b, the homogeneous (H) state with spatially uniform concentrations becomes linearly unstable against small perturbations when the reaction rates and diffusion constants satisfy certain conditions. This linear instability leads to spatial patterns that break the spatial translational symmetry of the underlying dynamics. Such mechanism is often called “Turing instability” or “diffusion-driven instability” [3], [9].

Many biological and physical systems can be described by RD models, for example, neuronal interactions of ocular dominance stripe formation [10], waves on the skin of the marine angelfish Pomacanthus [11], frequency-locking phenomena in a rotating spiral wave with external periodic forcing [12], interactions between zebrafish pigment cells [13], trans-membrane signal transduction [14], mussel population density patterns [15], etc.

Turing instability has long been regarded as the prototype mechanism for pattern formation in homogeneous RD systems [3]. Most theoretical studies on Turing patterns rely on linear stability analysis of the H state [16], [17] and its excitable wave numbers to establish general conditions for Turing instability. However, there are systems that can develop stable spatial patterns independent of the Turing instability [18], [19], [20], e.g., cell polarization responsible for cellular sensing and responsiveness [21], [22]. In these cases, stable patterns are not originated from small perturbations of the H state. However, except for Turing instability, other mechanisms of pattern formation in RD systems are not well understood. For example, in Fig. 1b, before Turing instability occurs (i.e., H state is stable), can large perturbations of H state develop stable spatial patterns? There are some existing works in RD systems supporting the coexistence of multiple H states [23], [24], [25]. For example, pattern formation can occur with equal diffusion coefficients in specific conditions where steady patterns could emerge from finite amplitude perturbations of a stable H state [23]. However, mechanisms of nonlinear instability for different RD models could be different. A systematic approach is desired to efficiently search for spatial stable and unstable patterns and reveal the underlying mechanism of RD systems.

In Turing’s original paper and most of the work afterwards, noise was not considered. However, dynamics of pattern formation in realistic biological systems are subject to strong noise due to the finite number of molecules involved in biochemical reactions. Fig. 1c is an example of pattern transition under noise. On a large time scale, the pattern with two peaks are more stable than the pattern with three peaks, hence accurately guarantees a specific spacing between concentration peaks. Given that spatial order under noisy environment is crucial in many biological processes [26], [27], an important question is how patterns maintain their spatial accuracy amid large spatial-temporal noise in the underlying biochemical reactions.

To address the two aforementioned fundamental problems in RD systems, i.e., nonlinear mechanism for pattern formation and robustness of spatial patterns, we introduce a solution landscape approach to systematically search the concentration functional space for steady-state solutions. Application of the solution landscape approach to generic RD models reveals a general nonlinear mechanism for pattern formation in the subcritical regime before the onset of the Turing instability. Next, based on the solution landscape framework, we develop a method to evaluate the stability of any given spatial pattern against noise by employing the concept of “action” from Freidlin-Wentzell large deviation theory [28], [29]. We apply the action-based stability analysis method to study a reversible three-species RD model. We show that the addition of the third species is crucial for stabilizing the spatial pattern against noise and significantly reduces fluctuations in spatial patterns despite its weak interactions with the other two chemical species.

## 2 Materials and methods

Two-species reaction-diffusion (RD) systems are simple but typical in generating spatial patterns. The deterministic dynamics of the two-species RD system can be generally formulated as:

(1) $$\begin{array}{cc} \frac{\partial u}{\partial t} & =D_{1}\Delta u+f\left(u,v\right),\\ \frac{\partial v}{\partial t} & =D_{2}\Delta v+g\left(u,v\right), \end{array}$$

where u and v are spatiotemporal variables representing concentrations of reactants. $D_{1}$ and $D_{2}$ are diffusion constants of two species, u and v, respectively. Functions f(u,v) and g(u,v) contain nonlinear terms representing biochemical reactions.

To find all possible stationary solutions, many numerical algorithms, *e.g.,* the eigenvector-following method [30], [31], the numerical polynomial homotopy continuation method [32], the deflation technique [33], and the WKBJ-based approach [34], have been developed. However, since these methods rely on suitable initial guesses, they may not find all the solutions and more importantly, they can not reveal the relationships between different solutions. Here, we introduce the solution landscape approach based on the generalized high-index saddle dynamics (GHiSD) to construct the solution landscapes of RD systems.

The solution landscape is a pathway map consisting of all stationary states and their connections [35], [36]. In an energy-based (or gradient) system, the solution landscape can be efficiently constructed by the high-index saddle dynamics (HiSD) [37] method combined with downward/upward search algorithms, and it has been successfully applied to Ginzburg–Landau model for phase transition [36], Landau–de Gennes model for liquid crystals [35], the Gross-Pitaevskii model for Bose–Einstein condensation [38], the Lifshitz–Petrich model for quasicrystals [39], etc. In this paper, we introduce GHiSD, which is modified from HiSD, in order to construct the solution landscapes for non-gradient systems.

Mathematically, we view stable stationary states as the sinks of the dynamic systems, and unstable stationary states are the saddle points. Here we adopt the index theory from infinite dimensional Morse theory [40] to give a rigorous mathematical description of topological invariants for saddle points of partial differential equation systems. The Morse index characterizes the nature of the nondegenerate saddle point, and an index-k saddle point (k-saddle) is a stationary state whose Jacobian matrix has exactly k eigenvalues with a positive real part [41]. From this perspective, a sink (referring to the fixed-point type in this paper) can be regarded as a 0-saddle point and two neighboring sinks are connected by a 1-saddle point (i.e., transition state). Every stationary solution is assigned an index. Particularly, a homogeneous solution with a nonzero index indicates the occurrence of Turing instability.

Morse theory provides a natural directed connection from higher-order saddles to lower-order saddles via the negative gradient flow in gradient systems [40], [42], [43]. This hierarchical structure according to Morse index forms a pathway map. In the non-gradient system, the solution landscape is a generalized pathway map aiming to reveal connections of all stationary states. The GHiSD algorithm combined with downward/upward search algorithms is applied to construct the solution landscape for the non-gradient system.

In Eq. (1), let $u={\left(u,v\right)}^{⊤}$, $F\left(u\right)={\left(D_{1}\Delta u+f\left(u,v\right),D_{2}\Delta v+g\left(u,v\right)\right)}^{⊤}$ and this RD system could be written as: $\frac{\partial u}{\partial t}=F\left(u\right)$, the formulation of GHiSD for a k-saddle (k-GHiSD) can be written as follows.

(2) $$\left\{ \begin{array}{c} \frac{\partial u}{\partial t}=\left(I-2\sum_{j=1}^{k}w_{j}w_{j}^{⊤}\right)F\left(u\right),\\ \frac{\partial w_{i}}{\partial t}=\left(I-w_{i}w_{i}^{⊤}\right)Jw_{i}-\sum_{j=1}^{i-1}w_{j}w_{j}^{⊤}\left(J+J^{⊤}\right)w_{i},\\ \;i=1,\ldots ,k. \end{array}\right.$$

The k-GHiSD involves a spatiotemporal variable u representing the concentrations of various substances and k direction variables ${\left\{w_{i}\right\}}_{i=1}^{k}$ approximating an orthonormal basis of the unstable subspace of the k-saddle. F is an operator on the spatiotemporal concentration function u, and $J\left(u\right)=\frac{\delta F}{\delta u}$ is the Jacobi operator.

The downward search algorithm is to apply GHiSD starting from a high-index m-saddle $u^{*}$ as a parent state to search for a low-index k-saddle (k<m). The initial searching position $u_{0}=u^{*}\pm \delta u$ is chosen to push the system away from $u^{*}$, and the pushing direction δu is along with a linear combination of (m−k) vectors whose negative eigenvalues have the smallest magnitudes chosen from ${\left\{w_{i}^{*}\right\}}_{i=1}^{m}$, which are the basis vectors of the unstable subspace (real Schur vectors) at $u^{*}$. When a complex eigenvalue with a negative real part includes, the pushing direction is along with the unstable subspace composed of the corresponding eigenvectors.

Also we have the upward search algorithm as an auxiliary to find higher-index saddles, especially the parent state(s) of the entire solution landscape. The combination of GHiSD and downward/upward search navigates the entire search up and down to construct the complete solution landscape containing the homogeneous state. The details of the GHiSD algorithm and downward/upward search in constructing the solution landscape are documented in Appendix A and Appendix B. The details of the spatial discretization in GHiSD of RD systems is provided in Appendix F.

## 3 Results

### 3.1 Pattern formation in supercritical turing regime

Here we will mainly focus on the 2-D Schnakenburg model [44], a minimal chemically realistic model that can give rise to Turing patterns [34], [45], [46], while our methodology can be applied in general RD models. The Schnakenburg model is described by PDEs in Ω=[0,L]×[0,L]:

(3) $$\begin{array}{ccc} \frac{\partial u}{\partial t} & = & \Delta u+\eta \left(a-u+u^{2}v\right),\\ \frac{\partial v}{\partial t} & = & d\Delta v+\eta \left(b-u^{2}v\right). \end{array}$$

The model corresponds to the following reactions

$$2u+v\overset{\eta }{\to }3u,\;⌀\overset{\eta a}{\to }u,\;u\overset{\eta }{\to }⌀,\;⌀\overset{\eta b}{\to }v.$$

Here u,v represent the density of activator and substrate(inhibitor) respectively and ⌀ represents the empty state. Parameter d denotes the relative diffusion constant of two species and η represents a relative balance between the diffusion and the chemical reaction. Parameters a and b are the constant speeds of two species produced in the domain uniformly respectively. The boundary condition is generally set as no-flux conditions for both species, i.e. $\frac{\partial u}{\partial n}{{|}_{\partial \Omega }=\frac{\partial v}{\partial n}|}_{\partial \Omega }=0$. Noted that the Schnakenberg model we used is non-dimensionalised PDEs and the parameter that truly determines the system’s diffusive behavior is $\sqrt{\eta }L$. An equivalent way to study the scale effect is to fix L=1 and change η in the experiment.

Many theoretical analyses have been done based on local linearization and excitable modes [47], [48] and have explained the influence of domain size of this model to generate Turing instability [49], [50]. Here, we use the solution landscape approach to map out all the steady state solutions in the parameter space.

We start with the unique, homogeneous stationary solution, i.e., H state, $\left(u_{0},v_{0}\right)=\left(a+b,b{\left(a+b\right)}^{-2}\right)$ in the Schnakenburg model Eq. (3). Using the linear stability analysis [16] (see details in Appendix C), the Morse index of H state can be explicitly calculated as Table 1.

**Table 1:** **Morse index of the H state with different**d**in the Schnakenburg model** ($\eta =200,L=1,a=\frac{1}{3},b=\frac{2}{3}$).

d	(0,44.6)	(44.6,47.4)	(47.4,58.4)	(58.4,74.9)	(74.9,+∞)
Index	0	2	3	5	7

The H state is a stable sink when d is less than a critical value $d_{0}=44.6$. When $d>d_{0}$, the H state losses its linear stability and stable spatial solutions emerge from the H state via pitchfork bifurcations, which is exactly the Turing mechanism. Two questions immediately present themselves: How many stable solutions are there in the supercritical regime ($d>d_{0}$)? More importantly, are there any stable solutions in the subcritical regime ($d<d_{0}$)? We address these two questions below by using the solution landscape approach.

**Fig. 2:**
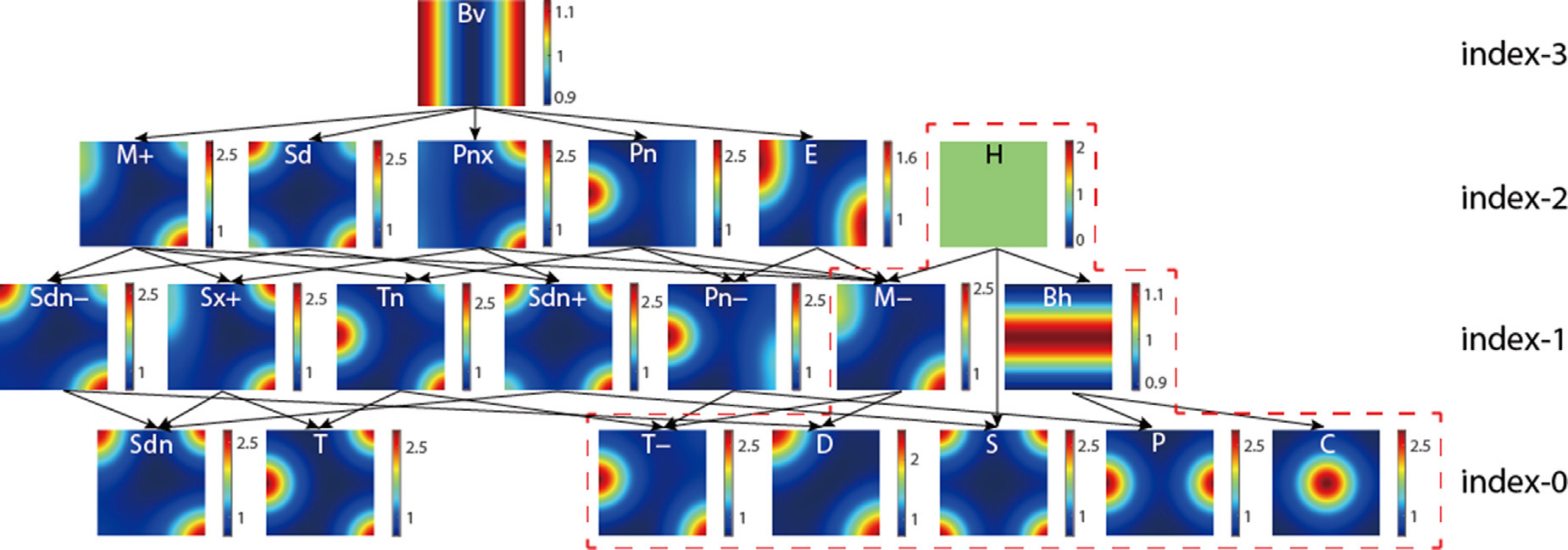
**Solution landscape of the Schnakenberg model in the supercritical regime** ($d=46>d_{0}$) **with**η=200 and L=1. Only u is shown in the figure (the same way in the following figures). When Turing instability occurs, H state is a saddle which generates multiple stable patterns. Pattern formation induced by Turing instability is highlighted in red dashed line area. Using the index-3 Bv as the parent state, the solution landscape shows more saddle points and new stable sinks. The nomenclature of stationary patterns is based on the shape and symmetry. The capital letter S (square), T (triangular), P (polar), C (circular), D (diagonal), B (banded), E (elliptical) and M (mixed) is to classify the shape roughly. The lowercase letter d (diagonal), x (x-axis symmetric), h (horizontal), v (vertical), n (not symmetric), + (some part additional), - (some part missing), m (minor in intensity) is used to mark the remaining or broken symmetry of patterns.

To find solutions in the supercritical regime, we start with the H state as the parent state at L=1. Here, we choose d=46 where the H state is a 2-saddle in the solution landscape. By applying the downward search algorithm, we can find all possible stationary states (1-saddles and stable sinks) that originate from the H state as shown in the region enclosed by the red dotted line in Fig. 2. The H state gives rise directly to one sink (S) and two 1-saddles M - and Bh, which subsequently give rise to T - & D sinks and P & C sinks, respectively. Note that the S, P and C states correspond to the same sink due to the symmetry of the pattern and boundary conditions. We validate this result by direct simulations of Eq. (3) using random perturbations of the H state (see details in Appendix D).

Although multiple sinks are generated from the H state through Turing instability, it is unclear whether it is the only way to generate stable solutions. To address this question, we apply the upward search starting from the known sinks to find other possible parent states. In particular, we pick T - as the starting state and use the upward search to find an upward pathway T - → Tn → Pn → Bv, in which Bv is a band-shape 3-saddle that has a higher index than that of H. Using Bv as the new parent state, we are able to compute a complete solution landscape shown in Fig. 2. It shows that there exist more 2-saddles (e.g. M+, Sd, Pnx, Pn and E) other than H, which connect new 1-saddles and then two new sinks, Sdn and T. Both these new sinks are easily found by using downward search through downward pathways from Bv, for example, Bv → M+ → Sx+ → Sdn and Bv → Pn → Tn → T. However, there is no pathway connecting H to Sdn and T, indicating that neither Sdn nor T can be generated by Turing instability.

### 3.2 Pattern formation in subcritical turing regime

**Fig. 3:**
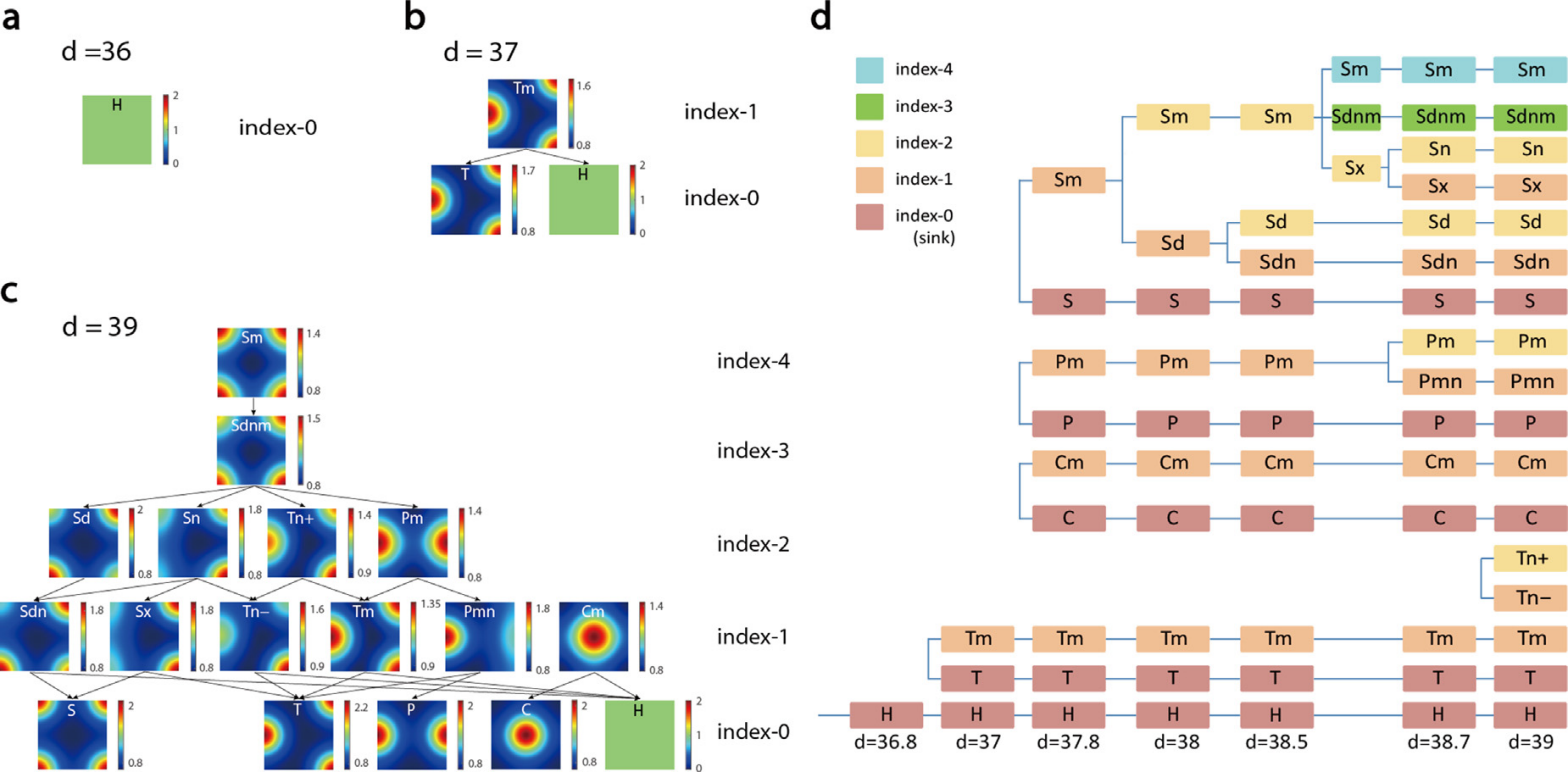
(a-c) Solution landscapes in the subcritical regime ($d<d_{0}=44.6$) with the relative diffusion speed d=36 (a), 37 (b), and 39 (c) at η=200 and L=1. From (a) to (c), solutions become abundant though H state is stable. (d) Bifurcation diagram of stationary solutions with increasing d.

The appearance of Bv, whose Morse index is higher than H, suggests that there may exist other mechanisms besides Turing instability for pattern formation. This prompt us to investigate the solution landscape of the Schnakenburg model in the subcritical regime ($d<d_{0}=44.6$) where the H state is stable. We find that the stable H state is the only stationary state for d≤36 (Fig. 3a). As d increases to 37, a pair of stationary states, Tm (1-saddle) and T (stable sink), emerges via a saddle-node bifurcation (Fig. 3b). If d becomes larger, more stationary states can be identified by applying the GHiSD method. For example, when d=39, Sm is found to be a 4-saddle. Using Sm as the parent state, we construct the corresponding solution landscape in Fig. 3c. It shows that four inhomogeneous sinks (S, T, P and C) exist besides H. Although S, P and C can be generated by Turing instability in the supercritical regime when H loses its stability at d=46, the emergence of these three states in the subcritical regime is due to some other mechanisms. By applying GHiSD for different values of d in the subcritical regime, we obtain the bifurcation diagram (Fig. 3d), which clearly shows that there exist a series of saddle-node bifurcations that generates stable sinks (T, S, P and C) while H remains stable. Note that the stable sinks can arise by infinitesimal perturbation from the H state in the supercritical regime. In contrast, when H is a stable sink in the subcritical regime, even though there exist transition pathways connecting H to certain inhomogeneous stable sinks such as T, S and C, a finite perturbation is required to overcome the transition barrier determined by transitional 1-saddle points, namely Tm, Tn - , Sdn and Cm.

In Fig S1 in the Supplementary Information we compare the basins of attraction of sinks when d=39. By linearly combining stable sinks and study the asymptotic pattern, we find that inhomogeneous sinks have quite large basins of attraction, indicating that these sinks are non-negligible subcritical patterns before the onset of the Turing instability. We further test the space of stable perturbations for each sink when d=39 by adding additive Gaussian white noise $u\left(x\right)=\max(u^{*}\left(x_{ij}\right)+\epsilon \sigma \left(x_{ij}\right),0),1\leq i,j\leq 32$, on a 32×32 coarse grid of Ω=[0,1]×[0,1], where $\sigma \left(x_{ij}\right)∼N\left(0,1\right)$ and are independent for different (i,j). $u^{*}$ represents stable sinks P,T,S,C and H. The asymptotic behavior indicates whether the state has escaped from the original stable sink. The escape ratio as a function of the amplitude ϵ is shown in Fig S1(d). When the amplitude ϵ<1, transition can seldom occur (escape ratio <1%) between these five sinks under perturbation. Considering that the concentration of species v is 0.5∼0.7 in stable patterns, ϵ=1 is quite large in the coarse grid, thus every sink is stable against large perturbations. The amplitude for 10 % samples of perturbations to escape from original sinks (defined as $\epsilon_{0.1}$) is $\epsilon_{0.1}=1.2∼1.7$ for P,T,S and C. The amplitude for H is much bigger ($\epsilon_{0.1}>2$) and it is not shown in Fig S1(d). We can conclude that the basin of attraction for each stable state has a quite large threshold for Gaussian-type perturbations.

**Fig. 4:**
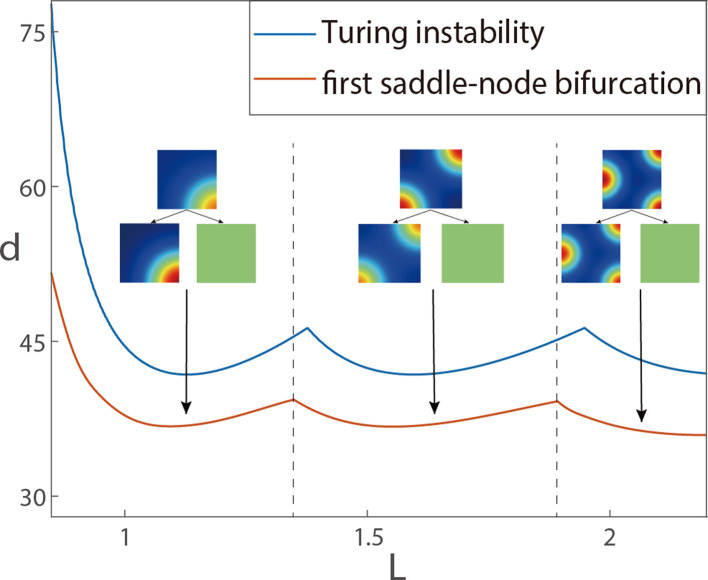
**Phase diagram for the first pattern formation over different**d**and square domain sizes**L**with**η=50**fixed**. The blue curve represents the Turing instability. The red curve represents the first saddle-node bifurcation, including three different saddle-node bifurcations (corresponding solution landscapes are inserted containing one 1-saddle and two sinks) separated by black dash lines.

To check whether the subcritical pattern formation depends on the system size, we also study the Schnakenburg model for different L (size of the square domain) and d. The phase diagram in the (L,d) space with η=50 fixed is shown in Fig. 4 where we plot the first saddle-node bifurcations with the corresponding solution landscapes as well as the Turing instability onsets for different values of L. It is clear from Fig. 4 that the first saddle-node bifurcation always occurs before Turing instability independent of system sizes. This nonlinear mechanism increases the parameter space where stable patterns exist beyond those originated from the Turing instability. We have also checked that different boundary conditions do not alter the main conclusions of this paper (see Fig S2 in the Supplementary Information for results with periodic boundary conditions).

Finally, to test the universality of our findings in the Schnakenburg model, we examine the solution landscape of the Gierer-Meinhardt model [2], [51]. The results in Fig S3 in the Supplementary Information support the existence of a general subcritical saddle-node bifurcation mechanism for pattern formation in RD systems.

### 3.3 Stability of spatial patterns against noise in solution landscape

The precise positioning of biomolecules is essential for many biological processes such as cell division and development. However, for biological systems, the size of the system and the number of molecules in the system are relatively small. As a result, dynamics underlying pattern formation in biological systems are subject to finite size effects and large stochastic fluctuations (noise). Therefore, understanding stability of various pattern forming states against noise is critical for understanding robustness of precise positioning in biological systems. For example, although a two-species RD system can generate spatial patterns, Murray & Sourjik recently highlighted the significance of an additional third species for enhancing the positional accuracy of Turing patterns in noisy biological systems [52].

**Fig. 5:**
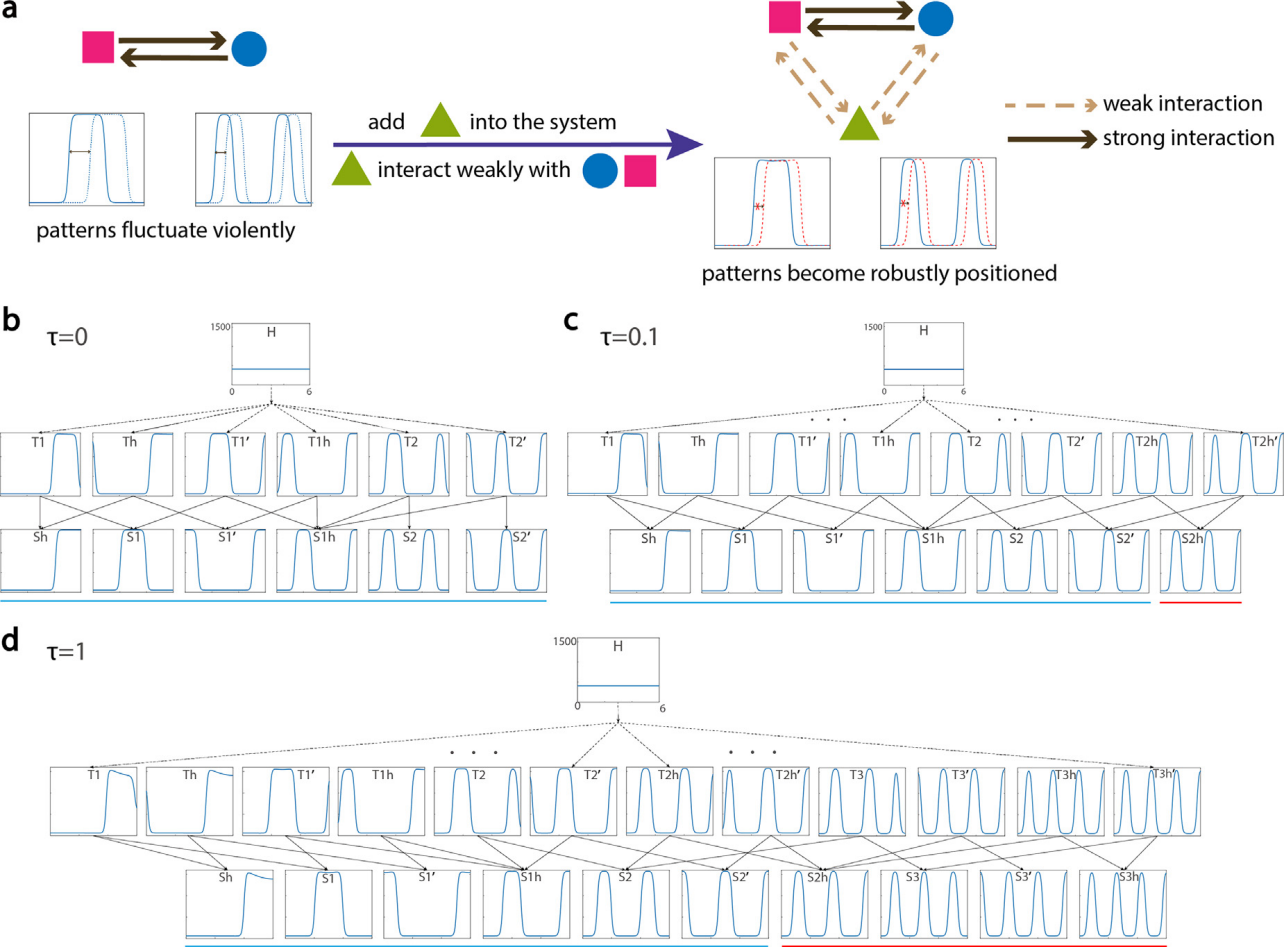
(a) Accurate positioning phenomena in three-species RD system. In the absence of the third species, patterns show considerable fluctuations. As long as the third species is introduced, although the interactions involved are very weak, patterns are positioned robustly.(b-d) Solution landscapes under parameter τ=0 (b), τ=0.1 (c) and τ=1 (d). Curves stand for spatial concentration of $X_{2}$ in the region Ω=(0,6). Sinks, 1-saddles and transition pathways are shown while high-index saddles are omitted. Blue underlines stand for the original stable patterns in τ=0 case while red underlines stand for newly formed stable patterns. “S” in the name of solutions represents for sinks and “T” for 1-saddles. The homogeneous state H is a 24-saddle in each solution landscape. The values of the coordinates are the same as those of the H states in (b-d). The number of the pattern’s peaks is used to name the pattern, and h denotes half peak. The apostrophe is used to distinguish patterns of the same number of peaks.

Here we consider a reversible three-species RD model in Fig. 5a proposed by Zhang et al. [53] where it was shown numerically that patterns formed in a two-species model are extremely sensitive to noise, and addition of the third species in the RD system can stabilize the patterns even with weak interaction strength (or equivalently small reaction rates). Reactions of this reversible model involve three linear reactions between each pair of three species $X_{1},X_{2},X_{3}$ and a nonlinear auto-catalytic reaction between $X_{1}$ and $X_{2}$:

$$X_{1}\underset{k_{21}}{\overset{k_{12}}{⇌}}X_{2}\text{,}\;X_{2}\underset{k_{32}}{\overset{k_{23}}{⇌}}X_{3}\text{,}\;X_{3}\underset{k_{13}}{\overset{k_{31}}{⇌}}X_{1}\text{,}\;X_{1}+2X_{2}\underset{{\tilde{k}}_{21}}{\overset{{\tilde{k}}_{12}}{⇌}}3X_{2}.$$

In the absence of noise, dynamics of three species’ concentration $u_{1},u_{2},u_{3}$ can be described by deterministic PDEs:

(4) $$\begin{array}{cc} \frac{\partial u_{1}}{\partial t}= & -{\tilde{k}}_{12}u_{1}u_{2}^{2}+{\tilde{k}}_{21}u_{2}^{3}+k_{21}u_{2}-k_{12}u_{1}\\ & -k_{13}u_{1}+k_{31}u_{3}+D_{1}\frac{\partial^{2}u_{1}}{\partial x^{2}},\\ \frac{\partial u_{2}}{\partial t}= & \;\;{\tilde{k}}_{12}u_{1}u_{2}^{2}-{\tilde{k}}_{21}u_{2}^{3}-k_{21}u_{2}+k_{12}u_{1}\\ & +k_{32}u_{3}-k_{23}u_{2}+D_{2}\frac{\partial^{2}u_{2}}{\partial x^{2}},\\ \frac{\partial u_{3}}{\partial t}= & -\left(k_{32}+k_{31}\right)u_{3}+k_{13}u_{1}+k_{23}u_{2}\\ & +D_{3}\frac{\partial^{2}u_{3}}{\partial x^{2}}, \end{array}$$

where x∈Ω=(0,6) and $u_{i}\left(i=1,2,3\right)$ satisfy no-flux boundary conditions. The reaction rate constants ${\tilde{k}}_{12},{\tilde{k}}_{21},k_{12},k_{21}$ between $X_{1}$ and $X_{2}$ are fixed as ${\tilde{k}}_{12}=1.67\times 10^{-5}$, ${\tilde{k}}_{21}=2.40\times 10^{-6}$, $k_{12}=0.5$, and $k_{21}=3.6$. The diffusion coefficients are chosen as $D_{1}=D_{3}=1.8$, $D_{2}=0.012$ such that the diffusion ratio $d=D_{1}/D_{2}=150$ is deep inside the Turing instability regime.

The reactions between the additional third species $X_{3}$ and $X_{1,2}$ are described by four reaction rates $k_{13}=k_{23}=0.0139\tau$, $k_{31}=0.0416\tau$ and $k_{32}=0.00139\tau$ where we introduce a scaling constant τ to control the interaction strength between $X_{3}$ and $X_{1,2}$. τ=0 representing the two-species model. Note that the interaction is weak even when τ=1 given the relatively small rates involving $X_{3}$. Our goal is to determine the stable solutions (sinks) and their stability as τ varies by using the solution landscape approach.

$X_{3}$ *introduces more stable patterns with higher wave numbers.* The solution landscapes corresponding to different values of τ(=0,0.1,1) are constructed. In Fig. 5, we show all 1-saddles and sinks as well as transition pathways connecting them. In the two-species model (i.e. τ=0 case), the solution landscape (Fig. 5b) has six sinks (underlined with a blue line) and six 1-saddles with relatively smaller wave numbers. As τ increases, more stable modes emerges in addition to the existing stable modes in the two-species model. These additional stable modes (1 for τ=0.1, 4 for τ=1, underlined with a red line respectively) have higher wave numbers as shown in Fig. 5c-d. The bifurcation diagram containing sinks and 1-saddles are documented in Fig S4 in the Supplementary Information. The emergence of these new patterns as τ increases can be understood in Fig S4(a), which shows the maximal real part of the eigenvalues (max(Reλ)) of 1-saddles (dotted line) and sinks (solid line) when τ increases from 0 to 1. Patterns with more than two peaks emerge via bifurcations when τ reaches some positive critical values when their corresponding max(Reλ) becomes positive. Three consecutive bifurcations highlighted by red circles in Fig S4a are shown in details in Fig S4b-d near their bifurcation points where newly generated stable stripe-patterns are also plotted in insets. Wave numbers of newly generated patterns increase $\frac{1}{2}$ for each consecutive bifurcation as τ is increased.

$X_{3}$ *enhances stability of the patterns.* Besides creating more stable patterns with higher wave numbers, the most important effect of the third species is to stabilize the two-species solutions (τ=0). As shown in Fig S4(a), as τ approaches 0, the maximal real parts of eigenvalues for the stable solutions are extremely small indicating poor positioning accuracy of the patterns in the presence of noise. However, the introduction of $X_{3}$ enhances the stability of sinks as max(Reλ) decreases significantly with the increase of τ.

To quantify the stability of the spatial patterns, we employ “action” based on Freidlin-Wentzell large deviation theory within the framework of solution landscapes. In particular, the action of a given path φ:[0,1]→H from φ(0) to φ(1) is defined as follows [54]:

(5) $$\hat{S}\left(\varphi \right)=\underset{T>0}{\inf}\underset{\psi \in {\bar{C}}_{\varphi }\left(0,T\right)}{\inf}S_{T}\left(\psi \right),$$

where ${\bar{C}}_{\varphi }\left(0,T\right)$ stands for all absolutely continuous functions on [0,T] whose image in the Hilbert space H is the path φ([0,1]). Action $\hat{S}\left(\varphi \right)$ is parametrization free. It can be regarded as the cost of changing the system from state φ(0) to state φ(1) along the path φ, and plays the role of energy cost in non-gradient systems. $S_{T}\left(\psi \right)$ is determined by the Lagrangian of the dynamics Eq. (4) (see details of the model’s action formula in Appendix E):

(6) $$S_{T}\left(\psi \right)=\left\{ \begin{array}{cc} \int_{0}^{T}L\left(\psi ,\dot{\psi }\right)dt & \;\;\text{if}\;\psi \;\text{is}\;\text{absolutely}\;\text{continuous}\;\\ & \;\;\;\text{and}\;\text{the}\;\text{integral}\;\text{converges,}\;\\ +\infty & \;\;\text{otherwise,}\; \end{array}\right.$$

Since pattern formation results in breaking of translation invariance in the spatially homogeneous system, the translation degree of freedom reflects the (approximate) degeneracy of solutions, and it corresponds to the least costly mode (the “soft” mode) in the system. Therefore, to evaluate the stability of the spatial patterns, we take the deviation Δ to be the spatial translation distance and compute the action S(Δ) along the path of deviation Δ numerically (see Appendix E for details).

**Fig. 6:**
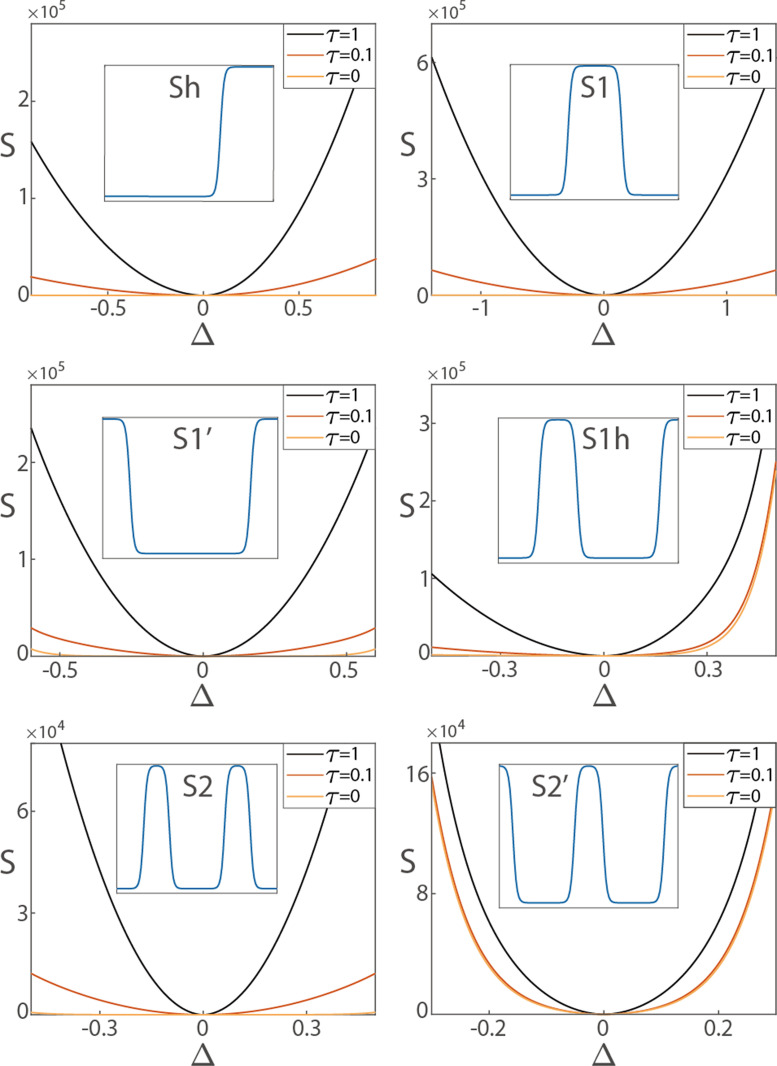
**The action-deviation** (S−Δ) **relations with**τ=0,0.1,1**for the 6 sinks emerged in the two-species case** (**see the six sinks in**Fig. 5(b)). The solution profiles for $X_{2}$: $u_{2}\left(x\right)$ are shown in the insets.

In Fig. 6, the action-deviation S−Δ relations for different values of τ=0,0.1,1 are shown for all six stable solutions (Sh, S1, S1${}^{\prime }$, S1h, S2 and S2${}^{\prime }$) in the two-species model (τ=0). It is clear that the action curve of each sink bends up as τ increases indicating the stabilizing effect of $X_{3}$. The S−Δ relations of the 6 sinks at τ=0 are shown in detail in Fig S5(a) in the Supplementary Information. With the exception of S2${}^{\prime }$, all the sinks show weak resistance to small shift indicating their weak stability against noise. In particular, Sh and S1 can shift freely with near zero action cost in a rather wide range of Δ, which implies that each of them is degenerate and lies in a one-dimensional solution manifold of Eq. (4).

To quantify the effect of $X_{3}$, we take the rightward shift (i.e. Δ≥0) as an illustration (since S−Δ curves for Sh, S1h are not axisymmetric, the shift towards two directions are different). As shown in Fig S5b, the action-deviation relations under different values of τ in log-log coordinates imply that S depends on Δ quadratically with its coefficient C(τ) depending on τ:

(7) $$S\left(\Delta \right)=C\left(\tau \right)\Delta^{2}.$$

To the leading order, we have:

(8) $$C\left(\tau \right)=\epsilon +\eta \tau +O\left(\tau^{2}\right),$$

where ϵ and η are different for different solutions. Table 2 shows ϵ and η for the six solutions that exist in the two-species model.

**Table 2:** **Expansion coefficient**ϵ**and**η**for six stable solutions**.

	Sh	S1	S1${}^{\prime }$	S1h	S2	S2${}^{\prime }$
ϵ	$<10^{-4}$	$<10^{-4}$	170	$2.4\times 10^{4}$	60	$2.7\times 10^{5}$
$\eta (\times 10^{5})$	4.7	3.1	5.9	3.1	2.3	3.8

The value of ϵ reflects the resistance to positional fluctuations when τ=0, and η characterizes the growth of such resistance as τ increases (larger C leads to smaller fluctuation and thus higher accuracy). Given that τ∼O(1) has already been regarded as weak coupling for $X_{3}$ in the system, the large value of the ratio $\frac{\eta }{\epsilon }$ reflects the strong effect of $X_{3}$ in stabilizing the sink solutions. The quadratic action gives rise to an effective Ornstein-Uhlenbeck process to describe the time evolution of shift Δ under noise:

$$d\Delta_{t}=-2\left(\epsilon +\eta \tau \right)\Delta_{t}dt+\sigma dW_{t}.$$

Thus $\Delta_{t}\to N\left(0,\frac{\sigma^{2}}{4(\epsilon +\eta \tau )}\right)$ as t→+∞, which clearly shows that the introduction of the third species (τ>0) increases the stability of the sink solutions. Without $X_{3}$, stability of the sinks is controlled by ϵ, which depends on the curvature of concentration functions near the boundary as shown in Appendix E. Therefore, for solutions Sh,S1,S1${}^{\prime }$ and S2 where ϵ is small due to their approximate translation invariance near the boundary, their stability is provided predominantly by $X_{3}$. Even for solutions S1h and S2${}^{\prime }$ with relatively larger ϵ, their stability is enhanced significantly by $X_{3}$ given the large value of η (see Table 2). Intuitively, fluctuations with long wave length, especially the shift of patterns are dampened by the non-local effect raised from a larger length scale of $X_{3}$ [53].

## 4 Discussion

In summary, we have developed an efficient numerical method to systematically construct the solution landscape of reaction-diffusion (RD) systems allowing us to find all possible stationary states connected to a homogeneous state and identify their relationships. By applying this general method to prototype RD models such as the Schnakenburg model and the Gierer-Meinhardt model, we discover a general nonlinear pattern formation mechanism wherein multiple stable heterogeneous states can emerge via saddle-node bifurcations and co-exist with the homogeneous state in the subcritical Turing regime before the onset of the linear Turing instability. However, the generality of this nonlinear instability across various RD models remains unclear. Specifically, there exists no unified criterion to determine under what conditions the parameter region of linear instability for an arbitrarily-designed RD system is encompassed within its region of nonlinear instability. Also, models with multiple H states may have complicated pattern formation before any H state loses its stability [23], [24], [25]. Our solution landscape approach is generic and can gain deeper insights in different RD models by systematically and efficiently calculating stationary patterns.

Furthermore, within the solution landscape framework, we developed a method to evaluate the stability of spatial patterns quantitatively by using action to describe the cost of deviation from the pattern caused by noise. To demonstrate its utility, this general method is applied to explain robust positioning of spatial patterns in a reversible three-species RD system with large intrinsic noise. Our method reveals the underlying mechanism for robust self-positioning even for very weak interactions of the third species with the other two chemical species in the system.

It is worth mentioning that we consider the robustness of deterministic patterns in deep region of Turing instability parameter space in the positioning of spatial patterns. The number of molecules within a correlation length of the system is very large. Therefore, spatial patterns are mainly the diffusion-driven type given by deterministic PDE models. However, fluctuation-driven stochastic “quasi-patterns” could emerge before Turing instability [55], [56], [57] and coexist with diffusion-driven patterns[58]. These stochastic “quasi-patterns” are not solutions to PDEs but may be important in the pattern positioning of real biological systems. The mechanism for robustness of “quasi-patterns” is different from the deterministic case and we leave it for future study.

As a generalization of high-index saddle dynamics (HiSD) developed to construct the solution landscape of gradient systems, the generalized high-index saddle dynamics (GHiSD) method presented in this study can be applied in generic PDEs including non-gradient systems to search for multiple stationary solutions and to uncover the pathways that connect these solutions. Besides the RD systems studied in this paper, GHiSD could also be used in convection-diffusion equations [59], [60], transport equations [61] and nonlinear Schrödinger equations [62]. However, for a model containing discrete variables (such as the Boolean type variable), HiSD and GHiSD fail to solve the minima and saddle points on the discrete energy landscape, because the execution of iteration in saddle dynamics depends on differentiation and the information of Hessian matrix.

From the theoretical and computational perspective, the solution landscape approach provides an efficient tool and a unifying view of pattern formation in RD systems. The general solution landscape approach could be instructive in studying pattern selection in synthetic systems [63], designing programmable reaction-diffusion systems [64], as well as understanding network robustness [65], from micro-scale systems such as cell regulation to macro-scale systems such as ecological networks [66].

## CRediT authorship contribution statement

**Shuonan Wu:** Writing – review & editing, Writing – original draft, Validation, Methodology, Investigation, Formal analysis. **Bing Yu:** Methodology, Investigation, Formal analysis. **Yuhai Tu:** Writing – review & editing, Writing – original draft, Supervision, Formal analysis. **Lei Zhang:** Writing – review & editing, Writing – original draft, Supervision, Project administration, Investigation, Funding acquisition, Formal analysis, Conceptualization.

## Declaration of competing interest

The authors declare that they have no conflicts of interest in this work.

## Appendix A GHiSD algorithm

The high-index saddle dynamics (HiSD) algorithm aims to find the index-k saddle point (k-saddle) starting from an initial guess $u_{0}$. It is initially designed for gradient systems [35]. Given a twice differentiable energy function E(u), where $u\in R^{n}$ is a vector variable, the HiSD algorithm is a pseudo-dynamics depicting the time derivatives $\dot{u}$ and $\dot{w_{i}}$. Here $w_{i}$ approximates the normalized eigenvector corresponding to the $i^{th}$ smallest eigenvalue, where i=1,…k. The HiSD for a k-saddle can be formulated as:

(A.1) $$\left\{ \begin{array}{cc} \dot{u} & =-\left(I-\sum_{i=1}^{k}2w_{i}w_{i}^{⊤}\right)\nabla E\left(u\right),\\ \dot{w_{i}} & =-\left(I-w_{i}w_{i}^{⊤}-\sum_{j=1}^{i-1}2w_{j}w_{j}^{⊤}\right)G\left(u\right)w_{i},\\ & \;i=1,\ldots k. \end{array}\right.$$

where G(u) is the Hessian matrix of E(u). A k-saddle $u^{*}$ along with its k unstable eigenvectors $w_{1},\ldots ,w_{k}$ will be attained, directly connected by $u_{0}$. Here $\langle w_{i},w_{j}\rangle =\delta_{ij}$ follows the orthonormal constraint for every t confirmed by Eq. (A.1).

For the non-gradient system $\dot{u}=F\left(u\right)$, we modify HiSD by replacing −G(u) with Jacobi operator J(u) and do some changes to the dynamics of $\left\{w_{i}\right\}$ to guarantee their orthonormality since J(u) is not self-adjoint. Then we get the generalized high-index saddle dynamics (GHiSD) for a k-saddle:

(A.2) $$\left\{ \begin{array}{c} \;\;\dot{u}=\left(I-2\sum_{j=1}^{k}w_{j}w_{j}^{⊤}\right)F\left(u\right),\\ {\dot{w}}_{i}=\left(I-w_{i}w_{i}^{⊤}\right)J\left(u\right)w_{i}\\ \;-\sum_{j=1}^{i-1}w_{j}w_{j}^{⊤}\left(J\left(u\right)+J^{⊤}\left(u\right)\right)w_{i},\;i=1,\ldots ,k. \end{array}\right.$$

In numerical implementation, the above dynamics are discretized in time using explicit Euler schemes on $u^{\left(m\right)}$ (m denotes the discrete time step of the pseudo-dynamics) and semi-implicit scheme for $\left\{w_{i}\right\}$. Since the Jacobian is usually expensive to compute, we adopt the dimer method to avoid evaluating the Jacobian explicitly.

(A.3) $$\begin{array}{c} \left\{ \begin{array}{c} u^{\left(m+1\right)}=u^{\left(m\right)}+\alpha \left(F\left(u^{\left(m\right)}\right)-2\sum_{j=1}^{k}\langle F\left(u^{\left(m\right)}\right),w_{j}^{\left(m\right)}\rangle w_{j}^{\left(m\right)}\right)\\ {\tilde{w}}_{i}^{\left(m+1\right)}=w_{i}^{\left(m\right)}+\beta J\left(u^{\left(m+1\right)}\right)w_{i}^{\left(m\right)},\;i=1,\ldots ,k,\\ \left[w_{1}^{\left(m+1\right)},\ldots ,w_{k}^{\left(m+1\right)}\right]=\mathrm{orth}\left(\left[{\tilde{w}}_{1}^{\left(m+1\right)},\ldots ,{\tilde{w}}_{k}^{\left(m+1\right)}\right]\right) \end{array}\right. \end{array}$$

where α and β are step sizes which are supposed to be sufficiently small to ensure the numerical stability. $J\left(u\right)w_{i}$ can be approximated as $(F\left(u+lw_{i}\right)-F\left(u-lw_{i}\right))/2l$ by a dimer method with a small constant l>0.

The initial unstable subspace of J, noted as $W^{u}\left(u\right)$, can be established by iteration of the orthonormal vector set $\left\{w_{1},\ldots ,w_{k}\right\}$ starting from k random initial vectors, which can be regarded as a power method of I+βJ:

(A.4) $$\left\{ \begin{array}{c} {\tilde{w}}_{i}^{\left(m+1\right)}=w_{i}^{\left(m\right)}+\beta J\left(u\right)w_{i}^{\left(m\right)},\;i=1,\ldots ,k\\ \left[w_{1}^{\left(m+1\right)},\ldots ,w_{k}^{\left(m+1\right)}\right]=\mathrm{orth}\left(\left[{\tilde{w}}_{1}^{\left(m+1\right)},\ldots ,{\tilde{w}}_{k}^{\left(m+1\right)}\right]\right) \end{array}\right.$$

In the Schnakenburg model, F and J can be formulated as:

(A.5) $$F\left(u\right)=\left(\begin{array}{c} \Delta u+\eta \left(a-u+u^{2}v\right)\\ d\Delta v+\eta \left(b-u^{2}v\right) \end{array}\right).$$

(A.6) $$J\left(u\right)\left(\begin{array}{c} f\\ g \end{array}\right)=\left(\begin{array}{c} \Delta f+\eta \left(2uv-1\right)f+\eta u^{2}\;g\\ -2\eta uvf+d\Delta g-\eta u^{2}g \end{array}\right),$$

where $u={\left(u,v\right)}^{⊤}$, f and g are arbitrary $C^{2}\left(\Omega \right)$ functions for spatial variables.

## Appendix B Downward and upward search

To identify all stationary states, we combine GHiSD with the following downward and upward search algorithms to construct the solution landscape.

To construct dynamic relations between two saddles, we pick a k-saddle $u_{0}$ as the initial state and search for (k−1)-saddles (or lower index saddles) using GHiSD. Once a new (k−1)-saddle is found, an arrow from $u_{0}$ to this saddle is drawn in the solution landscape to illustrate this relationship (see Fig. 2 for illustration). Based on (k−1)-saddles we can repeat the process above and construct a map revealing the evolutionary interrelationship between stationary states which we name the solution landscape. We call this kind of strategy searching from top to bottom the downward search strategy [35].

The downward search for k-saddles (k<m) can be numerically realized as follows. Given an m-saddle $u^{*}$ as the parent state, along with m unstable orthonormal vectors ${\left\{w_{i}\right\}}_{i=1}^{m}$ in $W^{u}\left(u\right)$ (the unstable subspace), we choose a linear combination of ${\left\{w_{i}\right\}}_{i=1}^{m}$, denoted as $\hat{w}$, and set the starting state for GHiSD as $u_{0}=u^{*}\pm \epsilon \hat{w}$ to give a small perturbation. Then a k-GHiSD starts at $u_{0}$ with k orthonormal vectors chosen from $\left\{w_{i}\right\}{}_{i=1}^{m}$ perpendicular to $\hat{w}$.

In the downward search from an m-saddle searching k-saddles by k-GHiSD, a preliminary attempt to choose $\hat{w}$ is to try: $\hat{w}=w_{i},\;i=1,\ldots m$, and set the initial state of k-GHiSD as $\left(u^{*}\pm \epsilon w_{i};w_{1},\ldots ,w_{k}\right)$, if i>k ; $\left(u^{*}\pm \epsilon w_{i};w_{1},\ldots ,w_{i-1},w_{i+1},\ldots ,w_{k+1}\right)$, if i≤k. Intuitively, this strategy makes the system to stay unstable in the chosen k directions but to roll off the original m-saddle in other (m−k) directions containing the perturbation direction $\hat{w}$. As a result, k-saddles can be found.

We can also use upward search when the parent state(s) is unknown or we need the information of higher index saddles (especially the parent states) based on the existing sinks or saddles. The search for m-saddles (m>k) starting from a k-saddle $u^{*}$ can be implemented as follows. Choose a stable direction $\hat{w}$ and m orthonormal initial directions including $\hat{w}$ at $u^{*}$. A typical choice for m-GHiSD upward search is $\left(u^{*}\pm \epsilon w_{m};w_{1},\ldots ,w_{m}\right)$.

It has been proved that $\left(u^{*},w_{1}^{*},\ldots ,w_{k}^{*}\right)$ is a linearly stable stationary point of the k-GHiSD if and only if $u^{*}$ is a k-saddle of the dynamical system[36]. Thus both the downward search and the upward search can reach saddles of the specified order k if k-GHiSD converges.

Combining the downward and upward search, we can construct the solution landscape of general dynamic systems using GHiSD. However, noted that in a general dynamic system, it is possible for a solution landscape to have multiple disconnected branches. In this case, the GHiSD method with downward and upward strategies is a local deterministic method operating within a single branch. Without appropriate initial guesses, the GHiSD method is unable to find some branches, in particular the branch made up of an isolated stationary point.

## Appendix C Linear analysis of the Schnakenburg model

The 2D Schnakenburg model [45], [46] in Ω=[0,1]×[0,1] can be formulated as:

(C.1) $$\begin{array}{cc} \frac{\partial u}{\partial t} & =\Delta u+\eta \left(a-u+u^{2}v\right),\\ \frac{\partial v}{\partial t} & =d\Delta v+\eta \left(b-u^{2}v\right). \end{array}$$

Here two species u and v satisfy the no-flux boundary condition. Parameter d denotes the relative dispersal speed of two species and η represents a relative balance between the diffusion and the chemical reaction. a and b are the constant speeds of two species produced in the domain uniformly respectively. The system has a unique homogeneous state (H for short), $\left(u_{0},v_{0}\right)=\left(a+b,\frac{b}{{\left(a+b\right)}^{2}}\right)$. Here we give the procedure of calculating Morse index of H in the presence of diffusion.

Linear analysis is implemented as follows. Let the small-perturbation of H be:

(C.2) $$\tilde{u}\left(x,t\right)=\left(\begin{array}{c} u-u_{0}\\ v-v_{0} \end{array}\right).$$

Now we look for the solutions of the form:

(C.3) $$\tilde{u}\left(r,t\right)=\sum_{k}c_{k}W_{k}\left(r\right)e^{\lambda t},$$

where $W_{k}\left(r\right)$ is a time-independent term satisfying a homogeneous Helmholtz equation with no-flux boundary condition:

(C.4) $$\nabla^{2}W+k^{2}W=0.$$

The solution of Eq. (C.4) takes the form:

(C.5) $$W=\left(\begin{array}{c} A\cos(m\pi x)\cos(n\pi y)\\ B\cos(m\pi x)\cos(n\pi y) \end{array}\right).$$

Substituting Eqs. (C.3), (C.5) into Eq. (C.1) and taking $a=\frac{1}{3},b=\frac{2}{3}$ and $\kappa =k^{2}=\pi^{2}\left(m^{2}+n^{2}\right)$, we get the secular equation:

(C.6) $$\left|\begin{array}{cc} -\kappa +\frac{\eta }{3}-\lambda & \eta \\ -\frac{4}{3}\eta & -\kappa d-\eta -\lambda \end{array}\right|=0.$$

Thus we have $\lambda_{1}+\lambda_{2}=-\left(\kappa d+\kappa +\frac{2}{3}\eta \right)<0$, and then a λ with positive real part exists if and only if $\lambda_{1}\lambda_{2}=\kappa^{2}d+\kappa \eta -\frac{1}{3}\eta \kappa d+\eta^{2}<0$. Finally we get the condition for Turing instability:

(C.7) $$d>\min_{\kappa \in K}\frac{\eta (\kappa +\eta )}{\frac{\eta }{3}\kappa -\kappa^{2}},$$

where $K=\{\pi^{2}\left(m^{2}+n^{2}\right)|m,n\in N\}$. Morse index of the H state is obtained by counting the number of possible (m,n) pairs in K with a fixed d satisfying:

(C.8) $$d>\frac{\eta (\kappa +\eta )}{\frac{\eta }{3}\kappa -\kappa^{2}},$$

By choosing $\eta =200,a=\frac{1}{3},b=\frac{2}{3}$, Morse index of the H state under different d is listed in Table. 1 of the text.

## Appendix D Direct simulation of the Schnakenburg model

Turing pattern can be obtained by using direct simulation of the Schnakenburg model. When the H state is unstable, we can add small perturbations to H as the initial guess, i.e., $u={\left(u_{0}+\delta u,v_{0}+\delta v\right)}^{⊤}$. Here the perturbations δu and δv can be generated as follows,

(D.1) $$\left(\begin{array}{c} \delta u\\ \delta v \end{array}\right)=\sum_{m,n\leq N}\left(\begin{array}{c} A_{m,n}\cos\left(m\pi x\right)\cos\left(n\pi y\right)\\ B_{m,n}\cos\left(m\pi x\right)\cos\left(n\pi y\right) \end{array}\right),$$

where N is a large integer and $A_{m,n},B_{m,n}$ are uniform distributions sampled from $\left[-\epsilon /\sqrt{N},\epsilon /\sqrt{N}\right]$, where ϵ is a small positive value to control the intensity of the disturbance.

We numerically solve Eq. (C.1) using the explicit Euler scheme in time and the finite volume scheme (see Appendix F) in space. By taking η=200,d=46 and different initial u, we obtained five sinks T - , D, S, P, C by solving Eq. (C.1) (see Fig. 2 of the text). These results are in consistence with the sinks derived in the solution landscape by using H as the parent state.

## Appendix E Action-deviation relation S(Δ)

For the field dynamics defined by PDE of the form: $\dot{\psi }=F\left(\psi \right)=\Delta \psi +f\left(\psi \right)$, Lagrangian in Eq. (6) is given by the Lagrangian density function $L(\psi \left(x\right),\dot{\psi }\left(x\right))$:

(E.1) $$L\left(\psi ,\dot{\psi }\right)=\int_{\Omega }L\left(\psi \left(x\right),\dot{\psi }\left(x\right)\right)dx$$

(E.2) $$L\left(\psi \left(x\right),\dot{\psi }\left(x\right)\right)=\frac{1}{2}{\left(\dot{\psi }-\Delta \psi -f\left(\psi \right)\right)}^{2}$$

In our three-species Turing model, Ω=(0,6). If the translation path φ(s),s∈[0,1] corresponding to deviation Δ (Δ≥0 for instance) is parameterized by:

(E.3) $$\begin{array}{cc} & \varphi_{\Delta }\left(s\right)\left(x\right)\equiv u_{s\Delta }\left(x\right)\\ & \equiv \left\{ \begin{array}{cc} \left(u_{1}\left(x-s\Delta \right),u_{2}\left(x-s\Delta \right),u_{3}\left(x-s\Delta \right)\right) & \text{if}\;s\Delta <x\leq 6\\ \left(u_{1}\left(0\right),u_{2}\left(0\right),u_{3}\left(0\right)\right) & \text{if}\;0\leq x\leq s\Delta \end{array}\right. \end{array}$$

we can establish an explicit formula of action for Δ shift along this path:

(E.4) $$\begin{array}{cc} S\left(\Delta \right)\equiv \hat{S}\left(\varphi_{\Delta }\right)= & \Delta \int_{0}^{1}∥u_{s\Delta }^{\prime }{\left(x\right)∥}_{L^{2}\left(\Omega \right)}\cdot {∥F\left(u_{s\Delta }\left(x\right)\right)∥}_{L^{2}\left(\Omega \right)}\\ & +{\langle u_{s\Delta }^{\prime }\left(x\right),F\left(u_{s\Delta }\left(x\right)\right)\rangle }_{L^{2}\left(\Omega \right)}ds \end{array}$$

where ${\langle \cdot \rangle }_{L^{2}\left(\Omega \right)}$ is the inner product under $L^{2}\left(\Omega \right)$ norm. This path $\varphi_{\Delta }\left(s\right),s\in \left[0,1\right]$ corresponds to a rightward shift process with constant speed v=Δ connecting the starting pattern $u_{0}\left(x\right)=\left(u_{1}\left(x\right),u_{2}\left(x\right),u_{3}\left(x\right)\right)$ and the end pattern $u_{\Delta }\left(x\right)$. Prolongation of left boundary of $u_{0}\left(x\right)$ is introduced artificially in Eq. (E.3) and obeys boundary conditions on the left (if Δ≤0, the boundary conditions are satisfied on the right).

For the translation path defined in Eq. (E.3), the second term of Eq. (E.4) vanishes. $∥u_{s\Delta }^{\prime }{\left(x\right)∥}_{L^{2}\left(\Omega \right)}$ and $∥F\left(u_{s\Delta }\left(x\right)\right){∥}_{L^{2}\left(\Omega \right)}$ in the integral above can be explicitly expressed by the starting pattern $u_{0}\left(x\right)=\left(u_{1}\left(x\right),u_{2}\left(x\right),u_{3}\left(x\right)\right)$:

(E.5) $$\begin{array}{cc} \;\;\;∥u_{s\Delta }^{\prime }{\left(x\right)∥}_{L^{2}\left(\Omega \right)}^{2}= & \int_{0}^{6-s\Delta }\sum_{i=1}^{3}{\left(\frac{du_{i}\left(x\right)}{dx}\right)}^{2}dx\\ ∥F\left(u_{s\Delta }\left(x\right)\right){∥}_{L^{2}\left(\Omega \right)}^{2}= & s\Delta \sum_{i=1}^{3}{\left(D_{i}\frac{d^{2}u_{i}\left(x\right)}{dx^{2}}{|}_{x=0}\right)}^{2}\\ +\Lambda & \sum_{i=1}^{3}{\left(D_{i}\frac{du_{i}\left(x\right)}{dx}{|}_{x=6-s\Delta -0}-0\right)}^{2} \end{array}$$

Where the last term in the expression of $∥F\left(u_{s\Delta }\left(x\right)\right){∥}_{L^{2}\left(\Omega \right)}^{2}$ is caused by boundary leaping of $\frac{du_{i}\left(x\right)}{dx}$ given no-flux boundary conditions which results in the singularity when calculating the Laplace term of F. This can be viewed as the penalty term due to violation of boundary conditions. Λ is the penalty factor. Large Λ makes the choice of prolongation less important (and we may simply consider the very simple prolongation in Eq. (E.3). Actually according to expansion:

(E.6) $$\begin{array}{cc} \frac{du_{i}\left(x\right)}{dx}{|}_{x=6-s\Delta } & \approx \frac{du_{i}\left(x\right)}{dx}{{|}_{x=6}-s\Delta \frac{d^{2}u_{i}\left(x\right)}{dx^{2}}|}_{x=6}\\ & =-s\Delta \frac{d^{2}u_{i}\left(x\right)}{dx^{2}}{|}_{x=6} \end{array}$$

we have

(E.7) $$\begin{array}{cc} S(\Delta ) & \approx \Delta^{2}\sqrt{\Lambda }{\left(\sum_{i=1}^{3}{\left(D_{i}\frac{d^{2}u_{i}\left(x\right)}{dx^{2}}{|}_{x=6}\right)}^{2}\right)}^{\frac{1}{2}}\int_{0}^{1}{∥u_{s\Delta }^{\prime }∥}_{L^{2}\left(\Omega \right)}sds\\ & \approx \frac{1}{2}\Delta^{2}\sqrt{\Lambda }{\left(\sum_{i=1}^{3}{\left(D_{i}\frac{d^{2}u_{i}\left(x\right)}{dx^{2}}{|}_{x=6}\right)}^{2}\right)}^{\frac{1}{2}}{∥u_{0}^{\prime }∥}_{L^{2}\left(\Omega \right)} \end{array}$$

Thus C in Eq. (7) roughly depends on the second derivative of solution on the boundary:

(E.8) $$C\approx \frac{1}{2}\sqrt{\Lambda }{\left(\sum_{i=1}^{3}{\left(D_{i}\frac{d^{2}u_{i}\left(x\right)}{dx^{2}}{|}_{x=6}\right)}^{2}\right)}^{\frac{1}{2}}{∥u_{0}^{\prime }∥}_{L^{2}\left(\Omega \right)}$$

The relationship between C and τ is shown in the dependence of solution $\left(u_{1}\left(x\right),u_{2}\left(x\right),u_{3}\left(x\right)\right)$ on τ. By treating τ as a small coefficient we could calculate the first order perturbation of solutions based on Eq. (4). Let

(E.9) $$\begin{array}{cc} u_{i}\left(x;\tau \right)=u_{i}^{0}\left(x\right)+\tau u_{i}^{1}\left(x\right)+\tau^{2} & u_{i}^{2}\left(x\right)+\cdots \\ & i=1,2,3 \end{array}$$

and $u_{i}^{0}\left(x\right),i=1,2$ is the two-species solution in the absence of $X_{3}$. $u_{3}^{0}\left(x\right)$ is a constant. The O(τ) term of Eq. (4) gives the equation to compute first order perturbation function $u_{i}^{1}\left(x\right),i=1,2,3$. This equation is a linear PDE:

(E.10) $$D\frac{d^{2}u^{1}\left(x\right)}{dx^{2}}=A\left(x\right)u^{1}\left(x\right)+c\left(x\right)$$

where $u^{1}\left(x\right)={\left(u_{1}^{1}\left(x\right),u_{2}^{1}\left(x\right),u_{3}^{1}\left(x\right)\right)}^{⊤}$ and satisfies no-flux boundary conditions. D is the diffusion coefficient matrix. A=A(x) and c=c(x) are:

(E.11) $$D=\left[\begin{array}{ccc} D_{1} & 0 & 0\\ 0 & D_{2} & 0\\ 0 & 0 & D_{3} \end{array}\right]$$

(E.12) $$A=\left[\begin{array}{ccc} {\tilde{k}}_{12}{\left(u_{2}^{0}\right)}^{2}+k_{12} & 2{\tilde{k}}_{12}u_{2}^{0}u_{1}^{0}-3{\tilde{k}}_{21}{\left(u_{2}^{0}\right)}^{2}-k_{21} & 0\\ -({\tilde{k}}_{12}{\left(u_{2}^{0}\right)}^{2}+k_{12}) & -(2{\tilde{k}}_{12}u_{2}^{0}u_{1}^{0}-3{\tilde{k}}_{21}{\left(u_{2}^{0}\right)}^{2}-k_{21}) & 0\\ 0 & 0 & 0 \end{array}\right]$$

(E.13) $$c=\left[\begin{array}{c} \frac{k_{13}}{\tau }u_{1}^{0}-\frac{k_{31}}{\tau }u_{3}^{0}\\ \frac{k_{23}}{\tau }u_{2}^{0}-\frac{k_{32}}{\tau }u_{3}^{0}\\ -\left(\frac{k_{13}}{\tau }u_{1}^{0}-\frac{k_{31}}{\tau }u_{3}^{0}\right)-\left(\frac{k_{23}}{\tau }u_{2}^{0}-\frac{k_{32}}{\tau }u_{3}^{0}\right) \end{array}\right]$$

According to Eq. (E.9), Eq. (E.8) can be formulated in the expansion of τ:

(E.14) $$C\left(\tau \right)=\frac{\sqrt{\Lambda }}{2}M\left(u^{0}\right)\times \left(1+\tau R\left(u^{0},u^{1}\right)+O\left(\tau^{2}\right)\right)$$

where

(E.15) $$\begin{array}{cc} \;M(u^{0})= & {\left(\sum_{i=1}^{3}{\left(D_{i}\frac{d^{2}u_{i}^{0}\left(x\right)}{dx^{2}}{|}_{x=6}\right)}^{2}\right)}^{\frac{1}{2}}{\left(\sum_{i=1}^{3}\int_{0}^{6}{\left(\frac{du_{i}^{0}}{dx}\right)}^{2}dx\right)}^{\frac{1}{2}}\\ R(u^{0},u^{1}) & =\frac{\sum_{i=1}^{3}D_{i}^{2}\frac{d^{2}u_{i}^{0}\left(x\right)}{dx^{2}}{{|}_{x=6}\frac{d^{2}u_{i}^{1}\left(x\right)}{dx^{2}}|}_{x=6}}{\sum_{i=1}^{3}{\left(D_{i}\frac{d^{2}u_{i}^{0}\left(x\right)}{dx^{2}}{|}_{x=6}\right)}^{2}}+\frac{\sum_{i=1}^{3}\int_{0}^{6}\frac{du_{i}^{0}}{dx}\frac{du_{i}^{1}}{dx}dx}{\sum_{i=1}^{3}\int_{0}^{6}{\left(\frac{du_{i}^{0}}{dx}\right)}^{2}dx}\\ & =\frac{\sum_{i=1}^{2}D_{i}^{2}\frac{d^{2}u_{i}^{0}\left(x\right)}{dx^{2}}{{|}_{x=6}\frac{d^{2}u_{i}^{1}\left(x\right)}{dx^{2}}|}_{x=6}}{\sum_{i=1}^{2}{\left(D_{i}\frac{d^{2}u_{i}^{0}\left(x\right)}{dx^{2}}{|}_{x=6}\right)}^{2}}+\frac{\sum_{i=1}^{2}\int_{0}^{6}u_{i}^{0}\frac{d^{2}u_{i}^{1}\left(x\right)}{dx^{2}}dx}{\sum_{i=1}^{2}\int_{0}^{6}u_{i}^{0}\frac{d^{2}u_{i}^{0}\left(x\right)}{dx^{2}}dx} \end{array}$$

The last step uses integration by parts and $u_{3}^{0}\left(x\right)=const.$. We get the final estimation for ϵ and η defined in Eq. (8):

(E.16) $$\begin{array}{cc} \epsilon & =\frac{\sqrt{\Lambda }}{2}M\left(u^{0}\right)\\ \eta & =\frac{\sqrt{\Lambda }}{2}M\left(u^{0}\right)R\left(u^{0},u^{1}\right) \end{array}$$

The ratio $\frac{\eta }{\epsilon }$ is exactly the linear coefficient $R(u^{0},u^{1})$. $R(u^{0},u^{1})\gg 1$ can be simply understood through the fact that:

(E.17) $$\begin{array}{cc} \left|\frac{d^{2}u_{i}^{0}\left(x\right)}{dx^{2}}{|}_{x=6}\right|\ll \left|\frac{d^{2}u_{i}^{1}\left(x\right)}{dx^{2}}{|}_{x=6}\right|, & i=1,2\\ \frac{d^{2}u_{i}^{0}\left(x\right)}{dx^{2}}{{|}_{x=6}\times \frac{d^{2}u_{i}^{1}\left(x\right)}{dx^{2}}|}_{x=6}>0, & i=1,2. \end{array}$$

Actually small second derivatives of $u^{0}$ stem from the almost free shift in two-species case. For example when u is the solution S1, $D_{1}\frac{d^{2}u_{1}^{0}\left(x\right)}{dx^{2}}{{|}_{x=6}\approx D_{2}\frac{d^{2}u_{2}^{0}\left(x\right)}{dx^{2}}|}_{x=6}\approx 10^{-10}$, but $u_{i}^{1}\left(x\right)$ is regularly solved from linear equation Eq. (E.10) thus $\frac{d^{2}u_{i}^{1}\left(x\right)}{dx^{2}}{|}_{x=6}∼O\left(1\right),i=1,2$. From Eq. (E.15) roughly we know $M\left(u^{0}\right)\propto \sum_{i=1}^{3}D_{i}\frac{d^{2}u_{i}^{0}\left(x\right)}{dx^{2}}{|}_{x=6}$ and $R\left(u^{0},u^{1}\right)\propto {\left(\sum_{i=1}^{3}D_{i}\frac{d^{2}u_{i}^{0}\left(x\right)}{dx^{2}}{|}_{x=6}\right)}^{-1}$. In this sense, ϵ depends linearly on the second derivatives of species’ concentration on the boundary, which vary widely from solution to solution and could be very small. But η is more or less the same for different sinks.

## Appendix F Spatial discretization in RD model

We adopt finite volume method to do the spatial discretization in PDE models with no-flux boundary conditions in this article. We give the 1-D version of the discretization to solve the problem described by Eq. (1).

Given the region Ω=[0,L], let $x_{\frac{1}{2}}=0$, $x_{N+\frac{1}{2}}=L$, $\Delta x=\frac{L}{N}$, $x_{j+\frac{1}{2}}=j\Delta x$ and $t_{m}=m\Delta t$, where j=0,…,N and m∈N. We integrate the first equation of Eq. (1) on $\left[x_{j-\frac{1}{2}},x_{j+\frac{1}{2}}\right]\times \left[t_{m},t_{m+1}\right]$ and after integration by parts we have:

(F.1) $$\int_{x_{j-\frac{1}{2}}}^{x_{j+\frac{1}{2}}}(u^{m+1}\left(x\right)-u^{m}\left(x\right))dx=\int_{t_{m}}^{t_{m+1}}(D_{1}\frac{\partial u}{\partial x}{|}_{x_{j-\frac{1}{2}}}^{x_{j+\frac{1}{2}}}+\int_{x_{j-\frac{1}{2}}}^{x_{j+\frac{1}{2}}}f\left(u,v\right)dx)dt,$$

where $u^{m}\left(x\right)≜u\left(x,t_{m}\right)$. Let ${\bar{u}}_{j}^{m}=\frac{1}{\Delta x}\int_{x_{j-\frac{1}{2}}}^{x_{j+\frac{1}{2}}}u^{m}\left(x\right)dx$, ${\bar{f}}_{j}=\frac{1}{\Delta x}\int_{x_{j-\frac{1}{2}}}^{x_{j+\frac{1}{2}}}f\left(u,v\right)dx$, we have:

(F.2) $$\begin{array}{cc} {\bar{u}}_{j}^{m+1}-{\bar{u}}_{j}^{m} & =\int_{t_{m}}^{t_{m+1}}(D_{1}\frac{\partial u}{\partial x}|{}_{x_{j-\frac{1}{2}}}^{x_{j+\frac{1}{2}}}+{\bar{f}}_{j})dt\\ & \approx \Delta t(\frac{D_{1}}{\Delta x}\frac{\partial u}{\partial x}|{}_{x_{j-\frac{1}{2}}}^{x_{j+\frac{1}{2}}}+{\bar{f}}_{j}){}^{m} \end{array}$$

The last step above we adopt explicit Euler discretization in time. Take the following approximation:

(F.3) $$\begin{array}{cc} \frac{\partial u}{\partial x}(x_{j+\frac{1}{2}}) & \approx \left\{ \begin{array}{c} 0,\;\text{if}\;j=0\;\text{or}\;N\\ \frac{u_{j+1}-u_{j}}{\Delta x},\;\text{otherwise} \end{array}\right.\\ {\bar{f}}_{j}^{m} & =f(u_{j}^{m},v_{j}^{m}) \end{array}$$

we get the discretization scheme of Eq. (1):

(F.4) $$\begin{array}{cc} \frac{U_{j}^{m+1}-U_{j}^{m}}{\Delta t} & =D_{1}\frac{U_{j+1}^{m}-2U_{j}^{m}+U_{j-1}^{m}}{{\Delta x}^{2}}+f\left(U_{j}^{m},V_{j}^{m}\right),\\ \frac{V_{j}^{m+1}-V_{j}^{m}}{\Delta t} & =D_{2}\frac{V_{j+1}^{m}-2V_{j}^{m}+V_{j-1}^{m}}{{\Delta x}^{2}}+g\left(U_{j}^{m},V_{j}^{m}\right),\\ & \;\;\;\;\;j=1,\ldots ,N \end{array}$$

and make conventions: $U_{0}^{m}=U_{1}^{m}$, $U_{N+1}^{m}=U_{N}^{m}$, $V_{0}^{m}=V_{1}^{m}$, $V_{N+1}^{m}=V_{N}^{m}$ to satisfy the no-flux boundary conditions on $x_{\frac{1}{2}}=0$ and $x_{N+\frac{1}{2}}=L$. The discretization scheme can be easily extended to 2D square region and is used in the first equation of Eq. (2).

## Appendix G Supplementary materials

Supplementary Data S1 Supplementary Raw Research Data. This is open data under the CC BY license http://creativecommons.org/licenses/by/4.0/ Supplementary Data S1
