# Supplementary material for: Solution landscape of reaction-diffusion systems reveals a nonlinear mechanism and spatial robustness of pattern formation
Source: Fundam Res. 2025 Oct 27;6(4):2137–48. doi: 10.1016/j.fmre.2025.10.006 (PMC13424414; doi:10.1016/j.fmre.2025.10.006)
Supplement: Supplementary Data S1 — Supplementary Raw Research Data. This is open data under the CC BY license http://creativecommons.org/licenses/by/4.0/ [file mmc1.pdf]

Supplementary materials: Solution landscape of reaction-diffusion systems reveals a nonlinear mechanism and spatial robustness of pattern formation

Shuonan Wu, Bing Yu, Yuhai Tu, Lei Zhang

Attraction basins of five sinks in Schnakenburg model in subcritical regime

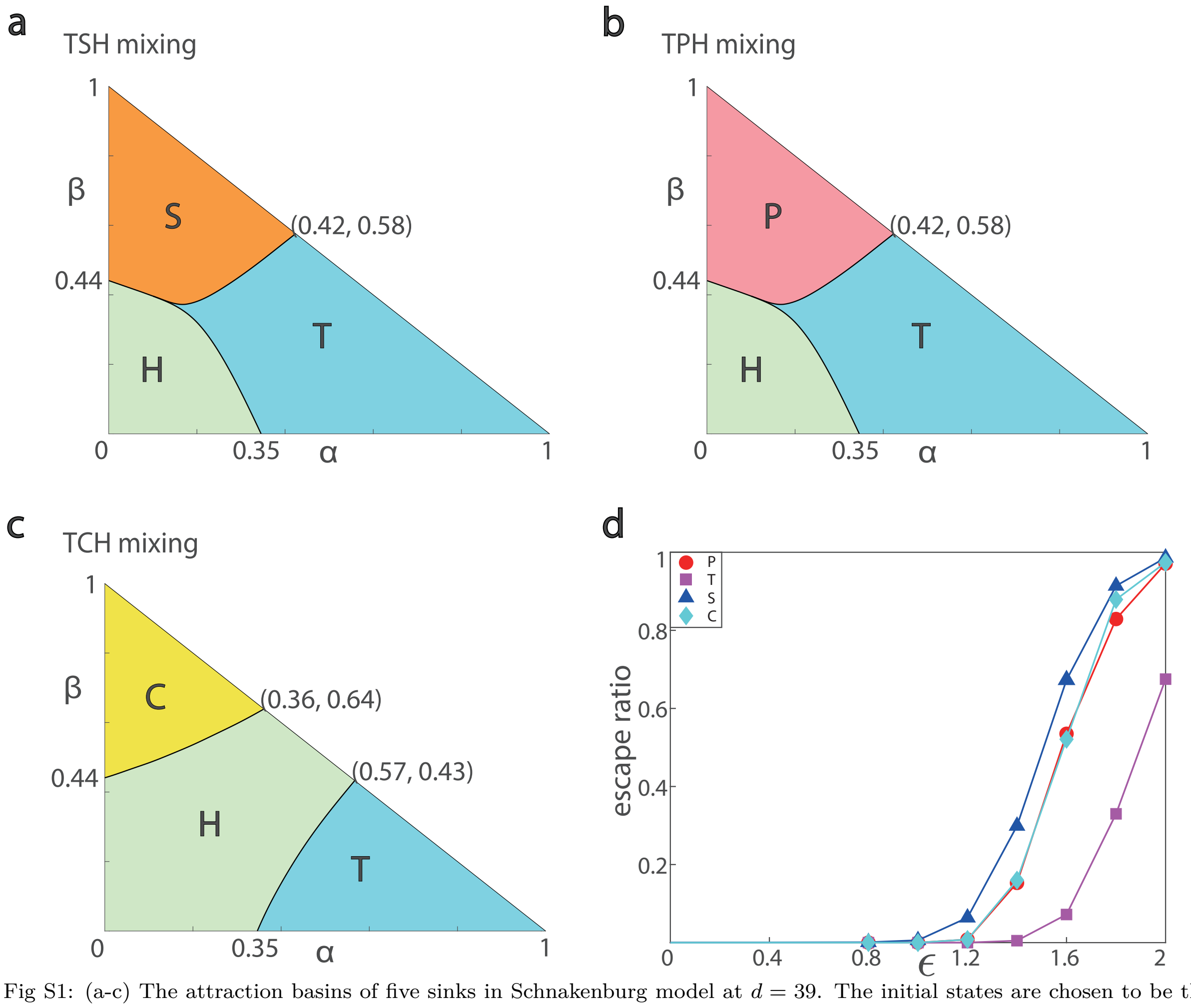

Fig S1: (a-c) The attraction basins of five sinks in Schnakenburg model at $d = 39$. The initial states are chosen to be the convex combination expressed as αT+βX+$(1-\alpha-\beta)$H, where X=S in (a), P in (b), and C in (c). (a) and (b) are exactly the same, and the attraction basin is separated into three regions in the domain $D = \{(\alpha,\beta)|\alpha \geq 0, \beta \geq 0, \alpha+\beta \leq 1\}$ by three critical lines. In (c), the attraction basins of C and T are separated by H. (d) The escape ratio after adding initial Gaussian-type perturbation at P, T, S and C as a function of perturbation amplitude ϵ. H state is not shown in the panel because it has a rather large ϵ to escape. The simulation results are based on the statistics of 1,000 independent samples for each sink and each fixed ϵ.

The influence of boundary conditions

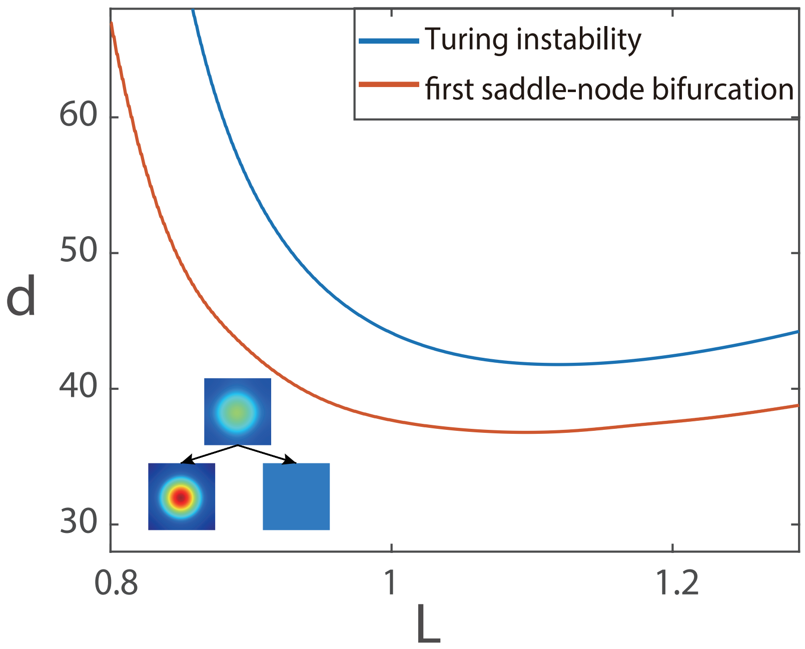

Fig S2: Phase diagram for different d and L with periodic boundary condition ($\eta = 204$).

Pattern formation in Gierer-Meinhardt model

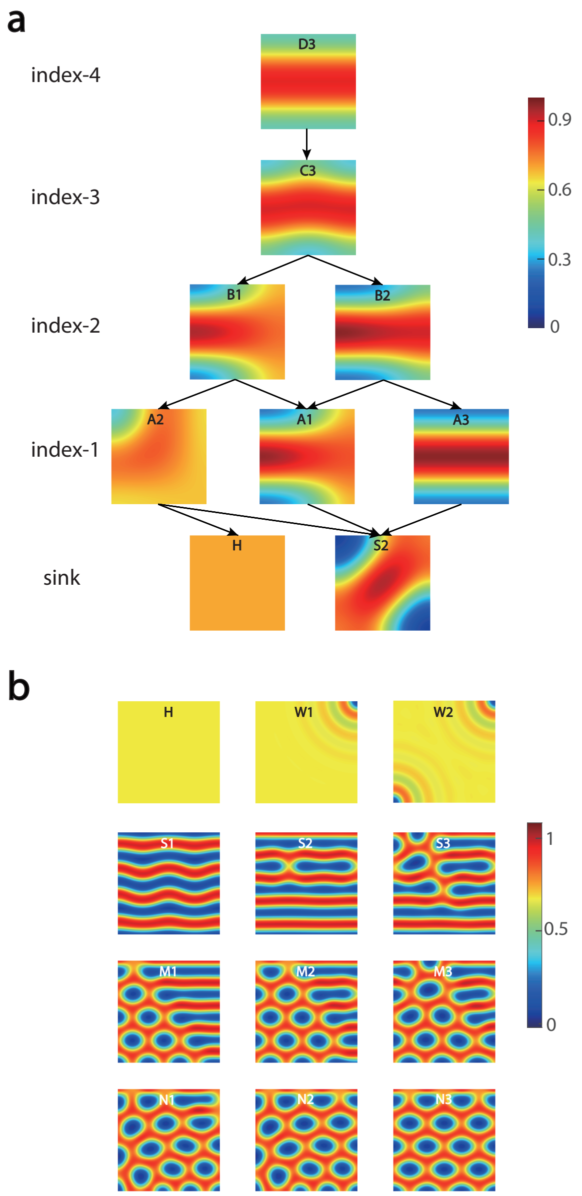

Fig S3: (a) Solution landscape of the Gierer-Meinhardt model in the 1×1 small square. (b) Some typical stable states in the solution landscape in the 5×5 square. Patterns of multiple shapes emerge when the system size becomes larger. The Gierer-Meinhardt model used here reads $\frac{\partial u}{\partial t} = a - bu + \frac{u^2}{v(1+cu^2)} + D_u \Delta u$, $\frac{\partial v}{\partial t} = u^2 - v + D_v \Delta v$ with no-flux boundary conditions, where $a = 0, b = 1.2, c = 0.4, D_u = 1 \times 10^{-2}$, and $D_v = 11 \times 10^{-2}$. The unique H state is stable under the given parameters.

Bifurcation diagram and eigenvalues of solutions in 3-species model

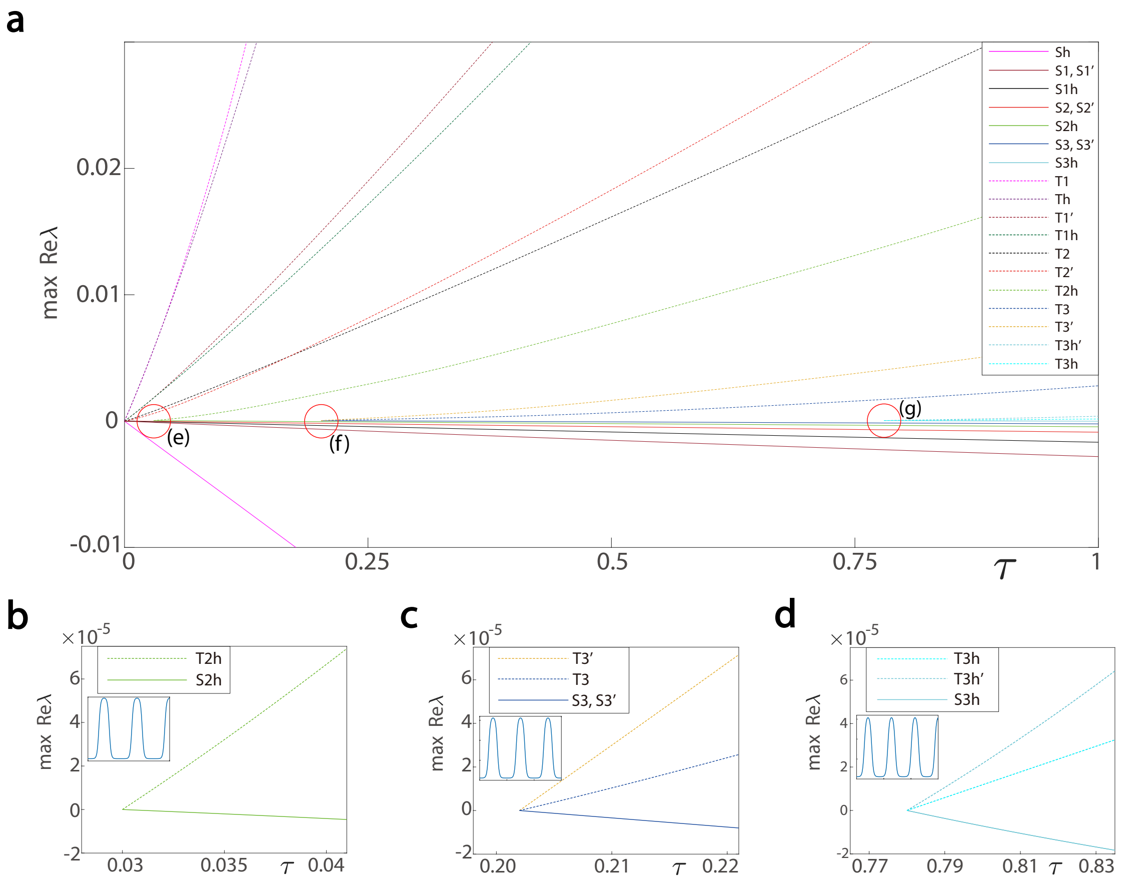

Fig S4: (a) The maximum value of the real part of eigenvalues of sinks and saddles (denoted by λ) under $\tau \in [0, 1]$. Dotted lines stand for 1-saddles and solid lines stand for sinks. Some sinks and saddles already exist at $\tau = 0$ while others emerge at positive τ. (b)-(d) Later-formed sinks and saddles through bifurcation.

Action-deviation relation at different τ

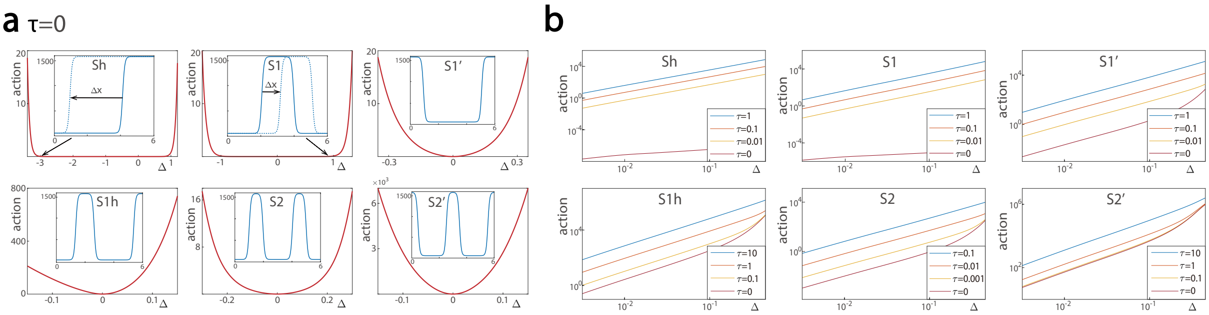

Fig S5: (a) Action-deviation ($S - \Delta$) relations (red lines) for 6 sinks which have already appeared in the 2-specie model ($\tau = 0$). The solution profiles for specie-2: $u_2(x)$ are shown in the insets. Note that the action-deviation curves for Sh and S1 sinks are flat near $\Delta = 0$ reflecting the approximate translational symmetry of the two sinks as shown in the corresponding insets. (b)Action-deviation relations of the six sinks in (a) for different values of τ. The slope ≈ 2 for every sink shows the quadratic relation. When τ is far from 0, the uniformly-spaced curves in each graph of Fig.5(b) indicate the validity of the linear expansion in Eq.8 in the text.
